# Syndromic surveillance in Vanuatu since Cyclone Pam: a descriptive study

**DOI:** 10.5365/WPSAR.2016.7.3.009

**Published:** 2011-12-19

**Authors:** George Worwor, Anthony David Harries, Onofre Edwin Merilles, Kerri Viney, Jean Jacques Rory, George Taleo, Philippe Guyant

**Affiliations:** a Ministry of Health, Port Villa, Vanuatu.; b WHO Country Liaison Office, Port Vila, Vanuatu.; c International Union Against Tuberculosis and Lung Disease, Paris, France.; d London School of Hygiene and Tropical Medicine, London, United Kingdom.; e The Pacific Community, Noumea, New Caledonia.; f Research School of Population Health, Australian National University, Canberra, Australia.

## Abstract

In 2012, Vanuatu designed and implemented a syndromic surveillance system based on the guidelines developed by the Pacific Community and the World Health Organization to provide early warning of outbreaks and other important public health events. Four core syndromes were endorsed for surveillance: acute fever and rash, prolonged fever, influenza-like illness and acute watery diarrhoea. In March 2015, Vanuatu was struck by Cyclone Pam, after which several important changes and improvements to the country’s syndromic surveillance were made. To date, there has been no formal evaluation of whether regular reports are occurring or that core syndromes are being documented. We therefore carried out a descriptive study in the 11 sentinel sites in Vanuatu conducting syndromic surveillance between July and December 2015. There was a total of 53 822 consultations which were higher in the first 13 weeks (*n* = 29 622) compared with the last 13 weeks (*n* = 24 200). During the six months, there were no cases of acute fever and rash or prolonged fever. There were cases with influenza-like illness from week 27 to 35, but no case was reported after week 35. Acute watery diarrhoea occurred in one or two cases per week during the whole study period. For these two core syndromes, there were generally more females than males, and about one third were children aged under 5 years. In conclusion, Vanuatu implemented changes to its new syndromic surveillance system from July to December 2015, although laboratory components had not yet been incorporated. The laboratory components are working in 2016 and will be the subject of a further report.

## Introduction

A central and historic responsibility for the World Health Organization (WHO) has been the management and control of the international spread of disease. To this end, International Health Regulations were formulated by WHO and adopted by the World Health Assembly in 1969. (*1*) In 2005, the World Health Assembly approved a second edition of the International Health Regulations in response to growth in international travel and trade and the emergence of the severe acute respiratory syndrome, the first global public health emergency of the 21st century. (*2*) Within this framework, Member States are mandated to collect information regarding public health events through surveillance activities and to assess the potential of these events to cause international spread of disease and possible interference with international travel and trade.

In recent decades, new diseases have emerged around the world that pose serious threats to regional and global security. The Asia Pacific Strategy for Emerging Diseases was developed in 2005, updated in 2010 and again in 2016 to meet the challenges of emerging diseases and acute public health threats in the Asia Pacific region. (*3*) From this strategy came a work plan for the Asia Pacific region with eight focus areas that included surveillance, risk assessment and response linked with accurate laboratory diagnosis. (*3*) In 2010, WHO and the Pacific Community (SPC) developed guidelines for the Pacific island countries and areas to design and implement a syndromic surveillance system to provide early warning of outbreaks and other important public health events so that immediate action could be taken to deal with epidemic infectious diseases. (*4*) Four core syndromes, along with case definitions and important diseases to consider, were endorsed for surveillance: acute fever and rash, prolonged fever, influenza-like illness and acute watery diarrhoea.

Vanuatu is a Y-shaped chain of islands located in the Pacific Ocean between the equator and the tropic of Capricorn. In 2012, syndromic surveillance based on the WHO PICTs guidelines was established and set up initially in three sentinel sites in the capital city, Port Vila. Five months later, the number of sentinel sites increased to eight. In March 2015, the island country was hit by Cyclone Pam. (*5*, *6*) There were several outbreaks and public health events after the cyclone that led to important changes and improvements in syndromic surveillance, including:

1. an increased number of trainings on syndromic surveillance from the SPC;
2. an increase in the number of sentinel surveillance sites to 11 by June 2015;
3. better appreciation from front-line health workers of the importance of syndromic surveillance;
4. a re-design of the sentinel site paper-based collection forms to record daily consultations (these data were not previously collected) and for ease in recording core syndromes;
5. introduction of a new weekly reporting template for use by the central unit, based on WHO surveillance reports; (*7*)
6. introduction of rapid diagnostic tests for malaria, dengue and leptospirosis; and
7. algorithms for sentinel sites to collect and send blood samples to the central unit for polymerase-chain-reaction (PCR) diagnosis which is done overseas.

By May 2015, and based on the surveillance system that was in place, the number of outbreaks and public health events had decreased in Vanuatu to the number before Cyclone Pam.

Since the introduction of the improvements to the syndromic surveillance system, there has been no formal evaluation of whether this system works for regular reports of patient consultations or counts of the four core syndromes. We therefore carried out a descriptive study in the 11 sentinel sites in Vanuatu conducting syndromic surveillance between July and December 2015 to determine the numbers of weekly consultations and the number of patients presenting with core syndromes of acute fever and rash, prolonged fever, influenza-like illness and acute watery diarrhoea along with data on gender and age group.

## Methods

### Study design

This was a descriptive study using already collected routine surveillance data.

### Setting

#### General setting

Vanuatu has 83 islands divided into six provinces with an estimated population of about 240 000. (*8*) It is classified as a lower middle income country according to the World Bank with an annual gross national income of US$ 1006–3975 per capita. (*9*) In each province there is a provincial hospital staffed by doctors and nurses, and the peripheral health care in the country is provided by 32 health centres, 99 dispensaries and 222 aid posts. Health care in the government sector and in the provincial hospitals is free of charge. There is one private health facility which is situated in Port Vila and serves a population of 10 000–15 000.

#### Syndromic surveillance at the sentinel sites

The surveillance unit in the Ministry of Health was established in June 2012 with the purpose of early detection and reporting of unusual cases and clusters of disease to the Ministry of Health and WHO and to respond rapidly to limit the impact of outbreaks. The 11 sentinel sites include six hospitals, one in each province, and five health centres located in five islands in three provinces selected because of remoteness, population sizes or damage from Cyclone Pam. The population sizes in the catchment areas of the sentinel sites varied from 2600 to 15 000. At each sentinel site, doctors and/or nurses record the number of outpatient consultations each day on specially designed forms. Any patient who has one or more core syndromes has details entered into the syndromic data form along with appropriate clinical and laboratory action taken (see **Table 1**). (*4*) Whenever possible, a clinical diagnosis is made, laboratory confirmation is attempted, treatment is given, isolation is recommended as appropriate and as agreed between staff of the sentinel sites and the central unit and notification is made to the director of public health and WHO in line with guidelines in the Pacific Outbreak Manual. (*10*)

**Table 1:** Core syndromes, case definitions, other important diseases to consider and laboratory actions

Core syndromes identified during syndromic surveillance	Case definition	Important diseases to consider	Laboratory action that should take place
Acute fever & rash	Sudden onset of fever* PLUS acute non-blistering rash	Measles, dengue, rubella, meningitis, leptospirosis	Blood sample sent to the central unit for transmission to New Caledonia for polymerase chain reaction investigation
Prolonged fever	Any fever* lasting 3 or more days	Typhoid fever, dengue, leptospirosis, malaria, other communicable diseases	Blood sample sent to the central unit for transmission to New Caledonia for polymerase chain reaction investigation
Influenza-like illness	Sudden onset of fever* PLUS: cough and/or sore throat	Influenza, other viral or bacterial respiratory infections	Naso-pharyngeal swab sent to the central unit for transmission to New Caledonia for polymerase chain reaction investigation only if the number of cases at sentinel sites exceeds a certain number
Acute watery diarrhoea	3 or more loose or watery stools in 24 hours	Viral and bacterial gastroenteritis, including cholera, food poisoning and ciguatera fish poisoning	Stool sample sent to the central unit for investigation at the central hospital, Port Villa, Vanuatu

* Fever is defined as 38 °C/100.4 °F or higher. If no thermometer is available, fever or chills reported by the patient or the caregiver are also acceptable.

Source: adapted from World Health Organization and Secretariat of the Pacific Community. (*4*)

#### Syndromic surveillance at the central unit

The consultations for one week at each of the 11 sentinel sites are sent routinely on Monday of every week to the central unit. If an alert threshold is exceeded in any of the four core syndromes at a sentinel site, the officer in charge of the central unit is immediately informed by telephone and initiates an in-depth investigation to confirm the alert. Syndromic data forms and laboratory samples, if available, are either collected by the officer in charge from nearby sentinel sites or sent to him by courier. The officer in charge then enters data for each core syndrome into the syndromic database. Data variables include the sentinel site, the name and contact details of the patient, age, sex, core syndrome, date of reporting of the core syndrome, clinical diagnosis, and, if available, details of the laboratory samples received at the central unit. If sentinel sites observe an unusual increase in the number of cases with a core syndrome, it is reported to the central unit within 24 hours and the central unit then recommends an investigation.

#### The syndromic surveillance situational report and follow-up action

The syndromic surveillance report is generated on a weekly basis from the central unit and sent in Vanuatu to all Ministry of Health cluster members, the national disaster management office and other government ministries. The report also goes to provincial health authorities who disseminate it to health centres, dispensaries and community-aid posts. An Epi-net response team then uses standardized procedures, as described in the Pacific Outbreak Manual, (*10*) to carry out field investigations. The syndrome data are shared weekly with WHO upon which the Pacific Syndromic Surveillance Report is generated and posted on PacNet. The syndromic surveillance reports highlight countries where thresholds for core syndromes are exceeded.

### Patient population

The study population included all patients presenting for consultation and identified with a core syndrome at 11 sentinel sites in Vanuatu between 1 July and 31 December 2015.

### Data variables, sources of data and data collection

Data variables included the sentinel site, the week of the year, the number of consultations in each week, the counts of the core syndromes, and for those with core syndromes the gender and the age (categorized as 0–4 years and 5 years and above). The source of data was the Excel electronic database maintained by the officer in charge of the central unit.

### Analysis and statistics

Data were single-entered from the Excel database into Epi Info^TM^ Version 7.0 (Centers for Disease Control and Prevention, Atlanta, GA, USA). A descriptive analysis was performed using absolute numbers, frequencies and proportions.

### Ethics

Permission for the study was given by the Ministry of Health as part of routine surveillance. Ethics approval for the writing and publication of the study was obtained from the Ethics Advisory Group, International Union Against Tuberculosis and Lung Disease (The Union), Paris, France. Patient consent was not required as this was anonymized secondary data.

## Results

**Fig. 1:**
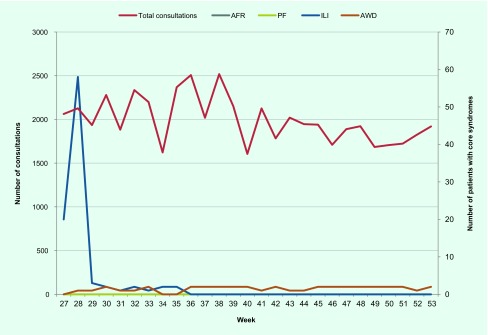
Weekly consultations along with the number of patients with core syndromes of influenza-like illness, acute watery diarrhoea, acute fever rash and prolonged fever in the 11 sentinel sites, Vanuatu, July to December 2015

Weekly consultations along with the number with core syndromes of influenza-like illness, acute watery diarrhoea, acute fever and rash, and prolonged fever between week 27 (1 July) and week 53 (31 December) 2015 are shown in **Fig. 1**. There was a total of 53 822 daily consultations which were higher in the first 13 weeks (weeks 27–40, *n* = 29 622) compared with the last 13 weeks (weeks 41–53, *n* = 24 200). During the six-month period, there were no cases of acute fever and rash or prolonged fever. However, there were cases with influenza-like illness and acute watery diarrhoea. Cases with influenza-like illness presented from week 27 to 35 and then stopped. There were generally one or two cases with acute watery diarrhoea for most of the weeks during the study period. Demographic characteristics of patients presenting with influenza-like illness and acute watery diarrhoea are shown in **Table 2**. There were generally more females than males, and about one third of the patients were children aged less than 5 years.

AFR = acute fever and rash, PF = prolonged fever, ILI = influenza-like illness, AWD = acute watery diarrhoea. Zero cases have been reported for AFR and PF.

**Table 2:** Demographic characteristics of patients presenting with influenza-like illness and acute watery diarrhoea

Characteristics	Influenza-like illness Number (%)	Acute watery diarrhoea Number (%)
All patients	91	40
Gender: Male Female	45 (49) 46 (51)	17 (42) 23 (58)
Age group in years: 0 – 4 5 and above	34 (37) 57 (63)	13 (32) 27 (68)

## Discussion

This study shows that the new syndromic surveillance system in Vanuatu set up to document the number of weekly consultations and the number of the four core syndromes worked with data being collated and produced in the electronic Excel database by the central unit. The main findings were a gradual decrease in weekly consultations in the fourth quarter of 2015 compared with the third quarter, reports of influenza-like illness in the third quarter that stopped completely in the fourth quarter, and one to two cases of acute watery diarrhoea that generally continued throughout the observation period. Thirty-seven per cent of patients with influenza-like illness and 32% of patients with acute watery diarrhoea were children aged less than 5 years.

An important finding was the large number of weekly consultations and yet the relatively small number of these presenting with one or more of the core syndromes. On reflection, this was probably due to several factors: 1) many of the focal officers in the sentinel sites who had been trained in syndromic surveillance were transferred to other facilities after Cyclone Pam leaving generally untrained personnel to do the reporting – hence it is likely that cases with core syndromes were missed; 2) poor telecommunication infrastructure after the cyclone especially with respect to mobile phones and e-mail access hindered reporting from peripheral sites to the centre; and 3) poor transportation also hindered reporting. These obstacles are in the process of being resolved, and for 2016, it is expected that reporting of core syndromes will improve.

There were some limitations to the study. The system reports only on those less than 5 years and those 5 years and above with no further categorization of this older age group. This needs to be modified. At least the age strata of 5–9 years, 10–19 years and over 20 years should be included as incidence of the syndromes may differ between these groups. In the second half of 2015, there were no operational systems in place for laboratory investigation and, therefore, no reports on recommended tests done, the time for samples to get to the central unit, the ease or difficulty of overseas sample testing or the time taken for results to get back to Vanuatu. Since 2016, however, the laboratory component has been started and gradually strengthened, although we have no collated data to report in this current study.

Since Cyclone Pam struck in March 2015, Vanuatu has implemented changes to its syndromic surveillance system. From July to December 2015, there were regular weekly reports of consultations along with reports of the number of people with one or more of the four core syndromes. Laboratory components had not yet been incorporated although work has been done in 2016 and will be the subject of a further report.
